# Traditionally used edible Solanaceae plants of Mizoram, India have high antioxidant and antimicrobial potential for effective phytopharmaceutical and nutraceutical formulations

**DOI:** 10.1016/j.heliyon.2021.e07907

**Published:** 2021-09-01

**Authors:** Laldinfeli Ralte, Usha Bhardwaj, Y. Tunginba Singh

**Affiliations:** a Department of Botany, Mizoram University, Aizawl 796004, Mizoram, India; b Department of Chemistry, Hunter College, CUNY, New York, NY 10065, USA

**Keywords:** Solanaceae, Ethnobotany, Bioactive phytochemicals, Antimicrobial, FT-IR, Mizoram

## Abstract

**Ethnopharmacological relevance:**

Solanaceae plants have been used as traditional medicines in Mizoram, India. This warrants the presence of therapeutic compounds and various bioactive phytochemicals in these plants, and characterizing their structures could lead to a possible focus for drug development.

**Aim of the study:**

Solanaceae plants are incredible sources of proteins and minerals; some even have high medicinal values which has been recognized traditionally. The present study was designed to explore and document the ethnobotany, phytochemical and mineral nutrient composition, antimicrobial properties, antioxidant potential and to identify functional groups from edible species of Solanaceae from Mizoram, India.

**Materials and methods:**

Field surveys and samples collection was conducted from Aizawl District, Mizoram, India. All the studied samples were extracted using Soxhlet apparatus for the analysis of bioactive compounds. The total phenol, total flavonoid and total anthocyanin contents were determined using standard methods. The antioxidant activities were measured using DPPH free radical scavenging, APX, CAT and SOD activities. The proximate analyses and mineral contents were determined by standard methods. The antibacterial potential was determined using the agar well diffusion method, and the functional groups were analysed using FTIR. All the results were reported as the mean ± standard deviation. The linear regression coefficient (R^2^) for total flavonoid and phenolic content with antioxidant activity was analysed using Graph Pad Prism Version 5. P-value < 0.05 was considered significant.

**Results:**

The phytochemical screenings showed the presence of alkaloids, tannins, flavonoids, terpenoids and saponins in all the samples. The highest total phenolic content was found in *Solanum anguivi* Lam. (29.51 mg GAE/g), and *Capsicum annuum* L. contained the highest total flavonoids (35.15 ± 0.03 mg/g). Proteins and carbohydrates contents were found to be the highest in *Solanum melongena* L. (28.49 mg/g) and *Physalis angulata* L. *(*35.64 mg/g) respectively. Elemental analysis showed the presence of Calcium (Ca), Copper (Cu), Iron (Fe), Manganese (Mn), Zinc (Zn), Potassium (K), Magnesium (Mg) and Sodium (Na) in high proportion in all the studied samples. All the plant extracts showed effective antibacterial activities against *Bacillus subtilis, Escherichia coli* and *Pseudomonas aeruginosa*. The Fourier Transformed Infra-Red Spectroscopy (FTIR) spectra revealed multiple functional groups in these plants species which could be used to identify bioactive compounds that can be subsequently utilized as herbal remedies for various ailments.

**Conclusion:**

Our findings suggest that a considerable amount of nutrients, biologically active and therapeutic compounds are present in the studied samples and these plants could be potential sources for new phyto-pharmaceutical and nutraceutical preparations.

## 1 Introduction

Plants are a rich source of nutrients and beneficial chemical compounds that can be used for the development of medicines. In recent years, traditional/herbal medicines are gaining special attention due to the increased concerns about safety, availability and negligible side effects as compared to synthetic medicines. In western countries, more than 40% of the pharmaceutical industries rely on medicinal plants (Deepashree et al., 2013). Plants also produce secondary metabolites that provide nutritional values and are involved in defence mechanisms against biotic and abiotic stresses to aid in their survival (El-Sayed et al., 2002). According to WHO, medicinal plants are the best sources for obtaining high-quality drugs and nearly 80% world's populations rely on traditional medicine for their health and well-being (Ellof, 1998) making them the preferred sources of compounds for pharmaceutical and healthcare products (Ivanova et al., 2005). High activity profile drugs have already been developed from biologically active compounds from medicinal plants. Crude extracts from medicinal plants are more biologically active than isolated compounds because of their synergistic effects (Jana and Shekhawat, 2010).

Solanaceae is one of the biggest plant families among the angiosperms with a great potential for providing food and medicinal security in the world. It comprises about 2300 species and is reported to be significant sources of phytochemicals and nutritional compounds in the pharmaceutical and food industry (D'Arcy, 1991; Eick, 2008). Some work on the anatomical and phytochemical characterization of *Physalis angulata* (Lea-Ferreira et al., 2020); elemental, proximate and phytochemical analysis of *Solanum incanum* (Burham, 2017); preliminary analysis and antimicrobial activity of *Solanum torvum* (Kalita et al., 2017); phytochemical analysis, antioxidant and anti-inflammatory of *Physalis peruviana* (Toro et al., 2014); phytochemical evaluation of *Solanum sp*. (Sundari et al., 2013); comparative morphological anatomical, cytological and phytochemical studies of *Capsicum sps*. (Wahua et al., 2013) and phytochemical screening, nutritional and toxicological analysis from leaves and fruits of *Solanum macrocarpon* (Dougnon et al., 2012) have been reported.

Mizoram, one of the states of the Northeastern (NE) region of India, lies in the Indo-Burman Biodiversity hotspot and is known for its high ethnic and cultural diversity. It has the highest tribal population (94.8%) among all the NE states (Lalramnghinglova and Jha, 1999). The Mizo tribes are mainly forest dwellers that rely on shifting cultivation for their livelihood. The majority of the population live in rural areas and most of their resources such as timber, food, medicinal plants etc. are obtained from the forest and hence they have a plethora of traditional knowledge on the uses of different plant products. However scientific data and documentation on ethnobotany, nutritional and phytochemicals of these plants is lacking. Considering the importance of Solanaceae species, the ultimate aim of this research is to improve the knowledge about these species. So, the study was designed to investigate the ethnobotanical uses; evaluate and analyse the bioactive phytochemicals, mineral nutrient compositions, antioxidant and antimicrobial potential of methanolic extracts of edible plants of Solanaceae from Mizoram. The outcome of the study will add to our understanding about the potential use of these plants in nutraceutical and pharmaceutical formulations.

## 2 Materials and methods

### 2.1 Plant material

**Figure 1:**
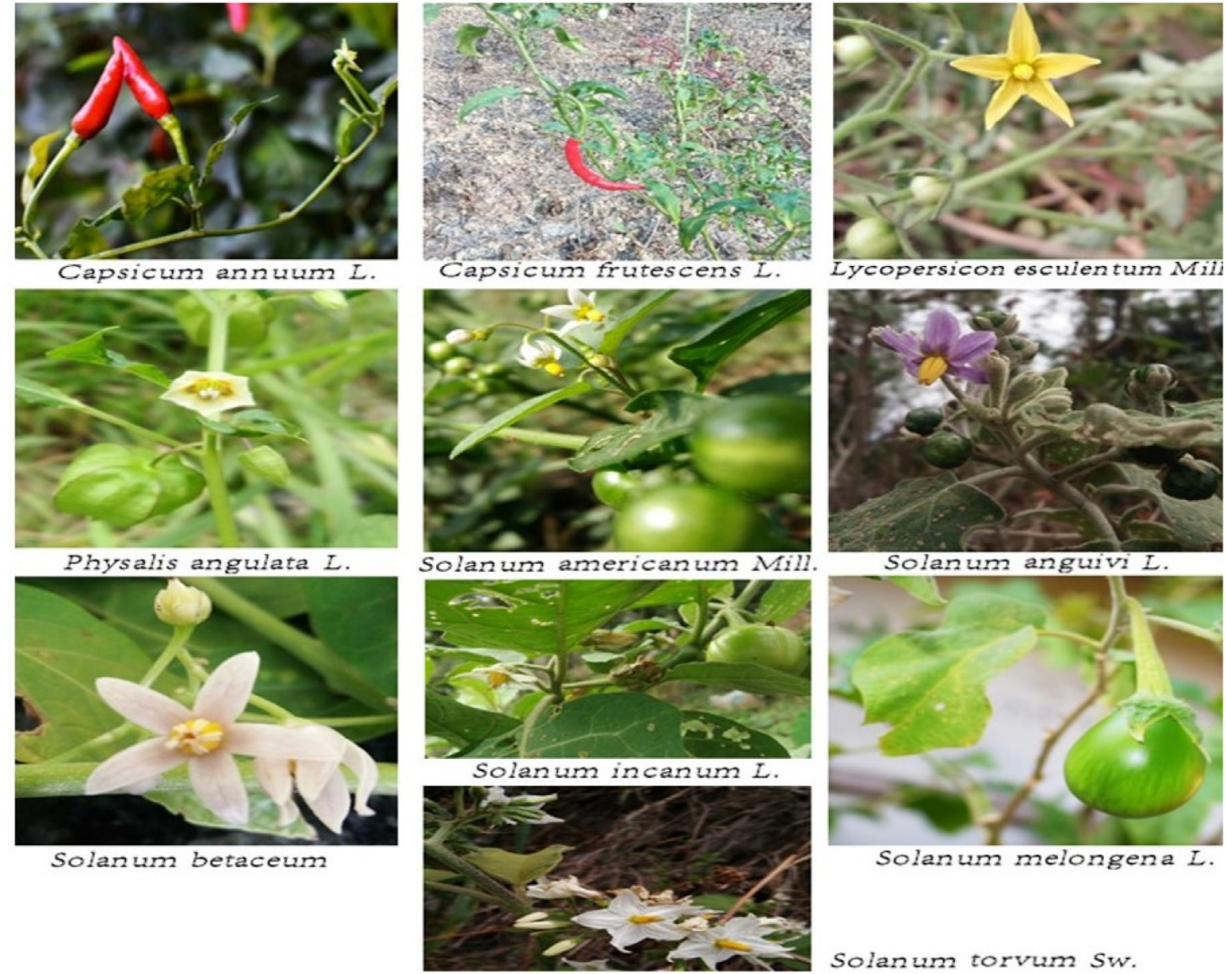
Solanaceae plants species used in the study.

Ten edible Solanaceae plants- *Capsicum annuum* L.*, C. frutescens* L*., Lycopersicon esculentum* Mill*., Physalis angulata* L*., Solanum americanum* Mill*., S. anguivi* Lam*., S. incanum* L*., S. melongena* L*., S. torvum* Sw*.* and *S. betaceum* Cav*.,* (Figure 1) regularly consumed by the Mizos, were collected from the wild, cultivated areas, roadsides and home gardens of Aizawl district of Mizoram. The collected plants were brought to the Department of Botany, Mizoram University for further analysis. Identification and confirmation of the collected specimen were done following published literature (Hooker 1872–1897; Singh and Singh, 2002). The specimens were also deposited in the Herbarium of the Department of Botany, Mizoram University. The ethnobotanical survey was conducted in the Aizawl district of Mizoram and was based on personal interviews with local herbal medicine practitioners and other knowledgeable local people and published literature.

### 2.2 Bioactive compounds analysis

#### 2.2.1 Samples preparation for extraction and phytochemical analyses

A 50g powdered sample of the edible parts from the selected Solanaceae plants, was extracted with 500mL of methanol using a Soxhlet apparatus for 25 cycles. The extract was then concentrated at 50 °C until it formed a paste. The concentration of each sample was adjusted to 100 μg/mL using methanol. Presence of various phytochemicals: alkaloids, saponins, flavonoids, tannins and terpenoids from these methanolic extracts were estimated using the procedure proposed by Nwankwo and Ukaegbu-Obi (2014).

#### 2.2.2 Determination of total phenolic content (TPC)

Total phenol was determined using Folin-Ciocalteu reagent method (Mc Donald et al., 2001) with slight modifications. A 100 μl plant extract sample was mixed with 0.1 ml Folin-Ciocalteu reagent (1N) and incubated at room temperature. Then, 5ml of Na_2_CO_3_ was added and incubated at room temperature for 30 min. Total phenolic content was determined using a UV-VIS spectrophotometer (Biospectrometer, Eppendorf, Germany) at 760nm. Gallic acid was used as standard and total phenol was expressed as gallic acid equivalent (mg/g of the extracted compound).

#### 2.2.3 Determination of total flavonoid content (TFC)

The total flavonoid content was determined using the Aluminium chloride calorimetric method (Chang et al., 2002) with some modifications. Briefly, 1ml methanolic extract was mixed with 1ml methanol, 0.5ml aluminium chloride (1.2%) and 0.5ml Potassium acetate (100mM) and incubated at room temperature for 30 min. The absorbance was measured at 415nm, and quercetin was used as standard. The total flavonoid content was expressed as quercetin equivalent (mg/g of the extracted compound).

#### 2.2.4 Determination of total anthocyanin content (TAC)

The total anthocyanin content was measured using a method proposed by Abdel-Aal and Hucl (1999). The methanolic extracts were mixed with acidified methanol (Methanol and 1N HCl, 85:15 v/v, pH1) and the absorbance was measured at 535nm against reagent blank. Cyanidin 3-Glucoside was used as a standard. Total anthocyanin content was calculated as Eq. (1):

(Equation 1) TAC (μg/g) = (A/ ε) × (vol/1000) × MW × (1/sample wt) × 10^6^

Where A is absorbance, ε is molar absorptivity of Cyanidin 3-Glucoside, vol is the total volume of anthocyanin extract and MW is the molecular weight of Cyanidin 3-Glucoside.

### 2.3 Evaluation of antioxidant activity

#### 2.3.1 DPPH radical scavenging activity

The antioxidant activity of the extract was determined with the 2,2-diphenyl-1-picrylhydrazyl (DPPH) radical scavenging method (Yan-Hwa et al., 2000). To 50μl of 10–100 μg/mL plant extract, 2 ml DPPH was added and kept in dark at room temperature for 30mins. Then, 1ml methanol and 2 ml DPPH was used as positive control while methanol solution was used as a negative control. Then the absorbance was measured at 517 nm. The percentage DPPH radical scavenging activity (%RSA) was calculated as Eq. (2):

(Equation 2) *%RSA = 100 × (absorbance of control – Absorbance of the sample)/ Absorbance of control*

#### 2.3.2 Catalase (CAT)

CAT activity was determined following Sunohara and Matsumoto (2004). Briefly, 0.1ml of the extract was mixed with 1.9ml of 25mM H_2_0_2_ in 50mM potassium phosphate buffer (pH 7). Then the absorbance was measured at 240nm. The enzyme activity was defined as the amount of H_2_0_2_ (mM) decomposed per minute.

#### 2.3.3 Ascorbate peroxidase (APX)

APX activity was determined by using Sunohara and Matsumoto (2004). About 2ml of the extract was mixed with 0.5ml of 100mM potassium phosphate buffer (pH 7), 0.5ml of 1mM ascorbic acid, 0.5ml of 0.4mM EDTA and 0.02ml of 10mM H_2_0_2._ Then, absorbance was measured at 290nm. The enzyme activity was defined as the amount of H_2_0_2_ (mM) decomposed per minute.

#### 2.3.4 Superoxide dismutase (SOD)

SOD activity was determined following McCord (2001). About, 3ml of the extract was mixed with 1.5M sodium carbonate, 0.1ml of 3mM EDTA, 0.2ml of 200mM methionine, 0.1ml of 2.25mM NBT, 1.5ml of 100mM potassium phosphate buffer, 0.95ml of distilled water and 0.5ml of extract. The tube without the extract was taken as control. The reaction was started by adding 0.1ml riboflavin (60uM) under light for 15mins. The absorbance was measured at 560nm and 1 unit of enzyme activity was defined as the quantity of enzyme which reduced the absorbance reading of samples by 50% in comparison with the control.

### 2.4 Nutrient determination

#### 2.4.1 Proximate analysis

For the estimation of protein and carbohydrates, 500 mg of edible parts were homogenized with phosphate buffer (50mM, pH 7.6). The extract was centrifuged at 8000 rpm for 10 min at 4 °C. The supernatant was then used for estimation of protein content following Lowry's method (Lowry et al., 1951) and Carbohydrate content using Hall (2007) method (Anthrone reagent) with glucose as a standard.

#### 2.4.2 Determination of mineral ion content

The standard protocol proposed by Moniruzzaman et al. (2014) was used for the determination of mineral ion contents in Solanaceae plants. One gram of air-dried sample was crushed and digested using Nitric Acid (HNO_3_) and Hydrogen Peroxide (H_2_O_2_) in a 5:1 ratio until it became crystal clear. The clear sample was cooled and diluted with distilled water to make up to 50ml. The diluted solution was filtered using a 0.2-micron membrane filter and analysed for detection of elements using Atomic Absorption Spectroscopy (Shimadzu AA-7000, Japan) and Microwave Plasma Atomic Emission Spectroscopy (4100 MP-AES, Agilent Technologies, USA).

### 2.5 Antimicrobial activity

The antimicrobial activities of the methanolic extracts from 10 Solanaceae species were tested against three bacterial strains- *Bacillus subtilis* ATCC11774, *Pseudomonas aeruginosa* ATCC9027 and *Escherichia coli* ATCC1229 using the agar well diffusion method.

### 2.6 FT-IR analysis

Functional groups present in the studied samples were identified using Fourier transformed infrared spectroscopy (Shimadzu IRAffinity-1S, Japan) for frequency ranging from 400-4000 cm^−1^ following the manufacturer's instruction.

### 2.7 Statistical analysis

All the results were reported as the mean ± standard deviation. The linear regression coefficient (R^2^) for total flavonoid and phenolic content with antioxidant activity was analysed using Graph Pad Prism Version 5. P-value < 0.05 was considered significant.

## 3 Results

### 3.1 Documentation of ethnobotanical uses

Ethnobotanical uses of 10 Solanaceae species used in the study are summarized in Table 1. Different plant parts are used for the treatment of various ailments as traditional medicines.

**Table 1:** Ethno-botanical uses of Solanaceae plants species used in the study.

Sl No	Species Name	Local Name	Part Used	Uses
1	*Capsicum annuum* L.	Hmarcha te	Leaves, Fruits	Fruits used as condiments, spices, improves digestion. Leaves prepared with fermented pork eaten as vegetables. Fruits and leaves juices applied to burn and snake bite. Fruits used as anti-haemorrhoidal, antiseptic, anti-rheumatic.
2	*Capsicum frutescens* L.	Hmarchapui	Leaves, Fruits	Fruits used as condiments, spices, improves digestion. Leaves prepared with fermented pork eaten as vegetables. Fruits leaves juices applied to burn and snake bite.
3	*Solanum betaceum* Cav*.*	Thingtomato	Fruits, Leaves	Fruits eaten as raw, cooked/roasted as vegetables. Also used in inflammatory painful disease, tonsils problem, liver problem. Leaves are heated on low flame and wrapped around the neck for sore throat.
4	*Lycopersicon esculentum* Mill.	Tomato	Fruits, Leaves	Fruits eaten as raw or cooked, also used as juice. Fruits used as skin care, treatment for sunburn. Leaves grinded in powder form are applied on spotted skin or leprosy spots.
5	*Physalis angulata* L.	Chal pangpuak	Fruits, Leaves	Fruits eaten as raw or cooked. Leaves are used as analgesic, antiseptic, asthma, diarrhoea. Fruits used for treatment of malaria, liver ailment, rheumatism, indigestion.
6	*Solanum americanum* Mill.	Anhling	Whole plant	Young shoot and leaves eaten as cooked. Decoction of whole plants are used as antispasmodic, anti-inflammatory blood purification, ulcers, anti-cancer, skin disease.
7	*Solanum anguivi* Lam.	Tawkte	Fruits, root, Leaves	Green fruit eaten as cooked or raw. Leaves are grinded and applied on skin disease, rash and spots. Fruits used as medicine for high blood pressure, asthma and stomach ache. Roots grounded to powder used as toothache, insect bites.
8	*Solanum incanum* L.	Samtawk	Fruits, roots, snake bites and wounds.	Green fruits eaten as cooked or raw. Fruits used as analgesic, medicine against high blood pressure, menstrual problem, sore-throat, stomach ache, liver problem, rheumatism, conjunctivitis. Roots or fruit rubbed on gums for toothache.
9	*Solanum melongena* L.	Bawkbawn	Fruits, Leaves, roots	Fruits cooked or roasted. Fruits used for lowering blood cholesterol level, high blood pressure, antihaemorrhoidal, antidote to poisonous mushrooms. Leaves as narcotics, skin disease, treatment for burns and bites. Decoction of leaves and roots used as toothache, bleeding and antiasthmatic.
10	*Solanum torvum* Sw.	Tawkpui	Fruits	Young fruits cooked or raw. Fruits used for treatment of fever, sore throats, stomach ache, chest pain. Used as antidiuretic, antidiabetic.

### 3.2 Analysis of bioactive compounds

The qualitative phytochemical analyses revealed the presence of alkaloids, flavonoids, saponins, tannins and terpenoids (Table 2) in all the plant extracts. The bioactive compounds; phenol, flavonoid and anthocyanin contents varied significantly among the samples (Table 3). TPC ranged from 9.87 to 29.51 mg GAE/g. Among the studied plants, *S. anguivi* had the highest phenolic contents while *S. torvum* had the lowest. Flavonoids exhibited noticeable variations among the plant extracts which ranged from 8.82 mg QE/g in *C. annuum* to 35.15 mg QE/g in *S. betaceum* (Table 3). The TAC varied from 0.069 to 0.91 mg/g in which *P. angulata* showed the highest and *C. annuum* had the lowest (Table 3).

**Table 2:** Phytochemical screening of Selected Solanaceae plants species.

Sl No.	Species Name	Parts tested	Alkaloids	Flavonoids	Saponin	Tannins	Terpenoids
1	*Capsicum annuum* L.	Fruits	+	+	+	+	+
2	*Capsicum frutescens* L.	Fruits	+	+	+	+	+
3	*Solanum betaceum* Cav*.*	Fruits	+	+	+	+	+
4	*Lycopersicon esculentum* Mill.	Fruits	+	+	+	+	+
5	*Physalis angulata* L.	Fruits	+	+	+	+	+
6	*Solanum americanum* Mill.	Leaves	+	+	+	+	+
7	*Solanum anguivi* Lam.	Fruits	+	+	+	+	+
8	*Solanum incanum* L.	Fruits	+	+	+	+	+
9	*Solanum melongena* L.	Fruits	+	+	+	+	+
10	*Solanum torvum* Sw.	Fruits	+	+	+	+	+

**Table 3:** Quantitative phytochemical analysis of Solanaceae plants species.

Sl. No	Species Name	Total Carbohydrate Content (mg/g)	Total Protein Content (mg/g)	Total Flavonoids Content (mg/g)	Total Phenolic Content (mg/g)	Total Anthocyanin content (mg/g)
1.	*Capsicum annuum* L.	19.12 ± 0.004	24.75 ± 0.005	35.15 ± 0.034	20.03 ± 0.006	0.069
2.	*Capsicum frutescens* L.	24.49 ± 0.009	22.95 ± 0.058	32.24 ± 0.001	19.14 ± 0.004	0.075
3.	*Solanum betaceum* Cav*.*	18.19 ± 0.012	16.93 ± 0.004	8.82 ± 0.002	12.30 ± 0.008	0.45
4.	*Lycopersicon esculentum* Mill.	25.27 ± 0.041	14.09 ± 0.004	16.56 ± 0.001	12.52 ± 0.007	0.91
5.	*Physalis angulata* L.	35.64 ± 0.011	17.05 ± 0.013	30.50 ± 0.002	21.57 ± 0.004	0.75
6.	*Solanum americanum* Mill.	16.48 ± 0.022	19.18 ± 0.038	23.20 ± 0.003	16.27 ± 0.005	0.5
7.	*Solanum anguivi* Lam.	26.95 ± 0.217	12.04 ± 0.007	16.56 ± 0.001	29.51 ± 0.004	0.38
8.	*Solanum incanum* L.	20.41 ± 0.011	21.76 ± 0.055	21.21 ± 0.002	14.95 ± 0.008	0.35
9.	*Solanum melongena* L.	18.54 ± 0.019	28.49 ± 0.058	19.66 ± 0.002	15.61 ± 0.006	0.25
10.	*Solanum torvum* Sw.	15.19 ± 0.012	6.1 ± 0.011	11.92 ± 0.037	9.87 ± 0.006	0.15

### 3.3 Enzymatic antioxidant activity

**Figure 2:**
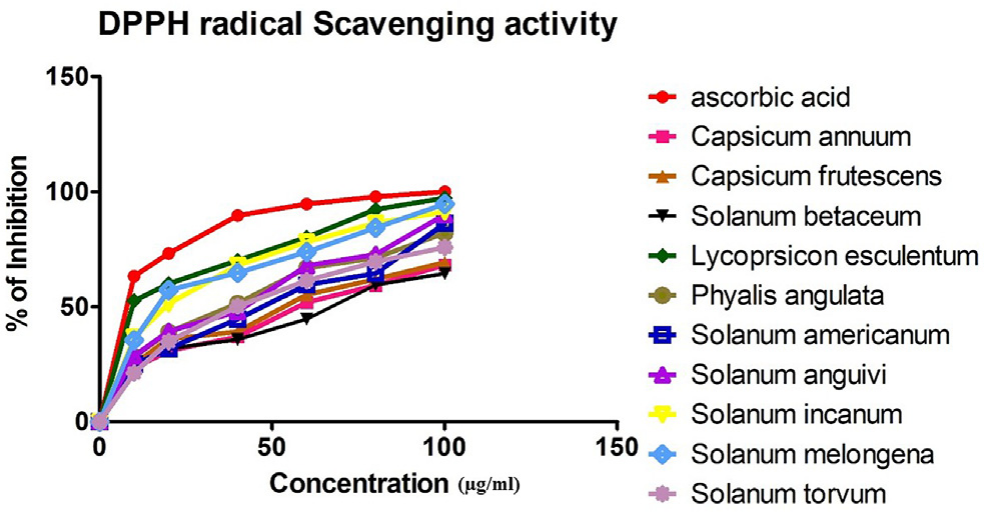
Antioxidant DPPH radical scavenging activity of Solanaceae species.

**Figure 3:**
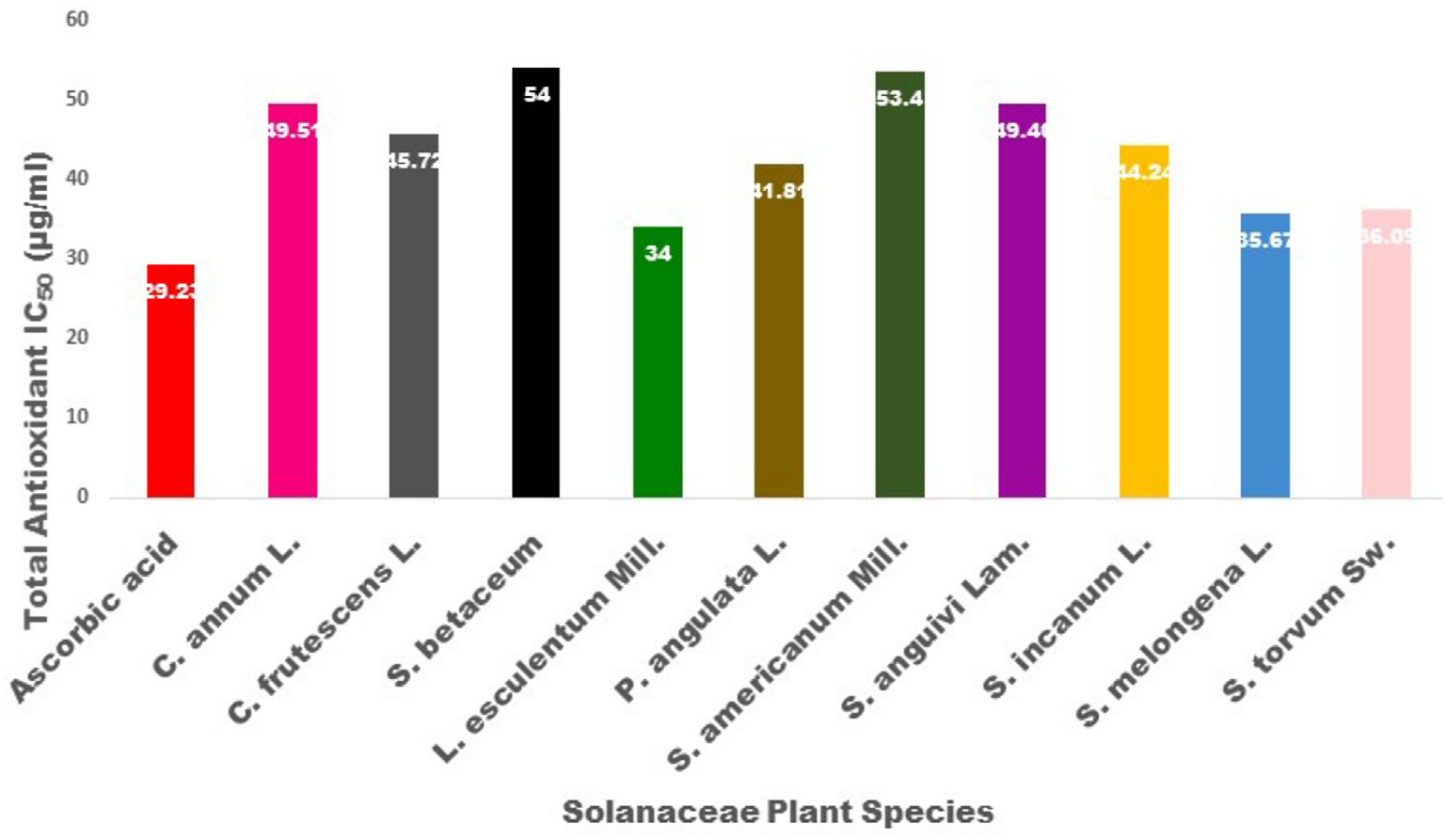
The DPPH antioxidant inhibition concentration (IC_50_) of Solanaceae plants.

The antioxidant capacity of plant extracts had significant scavenging activities on DPPH that increased with an increase in concentration (10–100 μg/ml) as shown in Figure 2. The IC_50_ value was calculated to determine the concentration of the sample required to inhibit 50% of free radicals and lower the IC_50_ value, higher the antioxidant activity (Li et al., 2009). The present study shows that the free radical scavenging activities of the extracts are concentration-dependent and comparable to ascorbic acid. The IC_50_ of the extracts and ascorbic acid observed in the study are detailed in Figure 3 and Table 4. Among the extracts, *L. esculentum* (34 μg/ml) showed the strongest IC_50_ value which was almost comparable to that of ascorbic acid. CAT, APX and SOD activities are shown in Table 4. The H_2_O_2_ decomposed per minute for catalase activity ranged from 0.32 mM to 6.31 mM. *S. anguivi* had the highest decompose rate at 6.31 mM H_2_O_2_ per minute while *S. melongena* decomposed the least amount of H_2_O_2_ per minute 0.32 mM H_2_O_2_). H_2_O_2_ decomposed per minute for APX activity ranged from 0.89 mM to 7.29 mM. *S. betaceum* decomposed 7.29 mM H_2_O_2_ per minute showing the highest APX activity and *C. annuum* (0.89mM H_2_O_2_ per minute) showed the lowest APX activity. The SOD activity of plant samples ranges from 0.23U to 1.63U. *L. esculentum* showed the highest SOD enzymatic activity while *S. torvum* possessed the lowest SOD enzymatic activity.

**Table 4:** Enzymatic antioxidant activity of Solanaceae plants species.

Species Name	Catalase (mM H_2_O_2_ decomposed/min	APX (mM H_2_O_2_ decompoed/min)	SOD (Unit)	Total Antioxidant IC_50_ (μg/ml)
*Capsicum annuum* L.	4.21	0.93	1.46	49.51
*Capsicum frutescens* L.	3.72	0.87	1.38	45.72
*Solanum betaceum* Cav*.*	4.2	7.94	1.32	54
*Lycopersicon esculentum* Mill.	0.89	0.98	1.63	34
*Physalis angulata* L.	0.52	1.24	0.94	41.81
*Solanum americanum* Mill.	5.61	1.4	0.23	53.4
*Solanum anguivi* Lam.	6.31	3.26	1.3	49.46
*Solanum incanum* L.	3.73	2.8	0.56	44.24
*Solanum melongena* L.	0.32	3.27	0.84	35.67
*Solanum torvum* Sw.	5.89	4.29	0.29	36.09
*Ascorbic acid*				29.23

### 3.4 Determination of nutrient composition

The nutrient composition of edible parts of Solanaceae plants is presented in Table 3. The protein content of the edible parts ranged from 6.1 mg/g in *S. torvum* to 28.49 mg/g in *S. melongena.* The carbohydrate content varied from 15.19 mg/g in *S. torvum* to 35.64 mg/g in *P. angulata.* The mineral compositions found in the study are presented in Table 5. High values of Na, Mg, Ca and K were found in all the samples and a considerable amount of Fe, Mn, Cu, Zn were also observed. The toxic mineral ions such as Pb and Ni were absent in the studied samples.

**Table 5:** Element analysis of Solanaceae Plants Species.

Sl No.	Species Name	Element Concentration (mg/kg)									
		Ca	Cu	Fe	Mn	Zn	K	Mg	Na	Ni	Pb
1	*Capsicum annuum* L.	1.39	0.019	0.23	0.05	0.072	1.4	0.02	1.4	0	0
2	*Capsicum frutescens* L.	1.68	0.02	0.41	0.05	0.17	0.9	6.56	1.71	0	0
3	*Solanum betaceum* Cav*.*	1.95	0.021	0.36	0.02	0.077	3.4	5.97	34.3	0	0
4	*Lycopersicon esculentum* Mill.	2.59	0.014	0.39	0.02	0.058	4.2	1	44.7	0	0
5	*Physalis angulata* L.	1.65	0.019	0.27	0.04	0.12	4	5.7	45.1	0	0
6	*Solanum americanum* Mill.	1.89	0.018	0.28	0.1	0.15	3.5	6.54	6.78	0	0
7	*Solanum anguivi* Lam.	2.79	0.02	0.26	0.04	0.12	2	4.05	2.23	0	0
8	*Solanum incanum* L.	2.34	0.039	3.24	0.12	0.11	2.2	1.5	34.1	0	0
9	*Solanum melongena* L.	2.73	0.039	0.43	0.12	0.12	1.3	2.29	9.24	0	0
10	*Solanum torvum* Sw.	5.46	0.045	0.4	0.1	0.1	2.3	1.14	43.4	0	0

### 3.5 Antimicrobial potential

The microbial growth inhibition of the methanolic extracts is summarized in Table 6. The antibacterial activities of extracts show strong effective inhibition activity against *Escherichia coli, Bacillus subtilis* and *Pseudomonas aeruginosa*. The maximum antibacterial activity was shown by *C. annuum* and the least by *S. torvum*.

**Table 6:** Antibacterial activity of solanaceae plants species.

Species Name	*Escherichia coli Inhibition Zone (mm)*			*Bacillus subtilis Inhibition Zone (mm)*			*Pseudmonas areuginosa Inhibition Zone (mm)*		
	20 mg/ml	40 mg/ml	60 mg/ml	20 mg/ml	40 mg/ml	60 mg/ml	20 mg/ml	40 mg/ml	60 mg/ml
Streptomycin (Positive control)	15.44 ± 0.58			20.44 ± 0.78			22.73 ± 0.69		
*Capsicum annuum* L.	7.33 ± 0.33	9.97 ± 0.54	11.9 ± 0.58	10.67 ± 0.34	14.67 ± 0.89	16.67 ± 1.76	9.33 ± 1.21	13 ± 0.57	14 ± 0.78
*Capsicum frutescens* L.	6.12 ± 0.46	8.2 ± 0.74	10 ± 1.31	9.45 ± 0.72	15.2 ± 0.42	16.62 ± 1.19	8.21 ± 0.67	11. 06 ± 1.12	15 ± 0.21
*Solanum betaceum* Cav*.*	3.45 ± 0.39	7.97 ± 0.89	10.89 ± 0.42	5.67 ± 0.38	8.88 ± 1.12	11.43 ± 0.47	6.33 ± 0.19	9.78 ± 0.49	12.22 ± 0.29
*Lycopersicon esculentum* Mill.	9.01 ± 0.54	11.74 ± 0.23	13 ± 0.36	8.5 ± 1.07	12.1 ± 0.22	14.2 ± 0.44	6.4 ± 0.56	9.2 ± 0.67	16.34 ± 0.67
*Physalis angulata* L.	5.03 ± 1.42	7.41 ± 0.44	10.2 ± 0.22	4.07 ± 0.33	6.51 ± 0.81	9.5 ± 0.61	5.6 ± 0.88	7.8 ± 1.41	10.7 ± 0.74
*Solanum americanum* Mill.	5.76 ± 1.12	7.45 ± 0.67	9.01 ± 1.21	7.22 ± 0.11	10.67 ± 0.96	12.45 ± 0.11	8.56 ± 0.68	11.33 ± 0.33	12.22 ± 0.29
*Solanum anguivi* Lam.	6.33 ± 0.33	8.22 ± 0.11	10.66 ± 0.48	7.66 ± 0.11	11.33 ± 0.44	14.55 ± 0.58	7.44 ± 0.56	9.27 ± 0.63	11.67 ± 1.02
*Solanum incanum* L.	4.27 ± 0.11	7.01 ± 0.41	7.92 ± 0.34	4.22 ± 0.64	8 ± 0.49	9.3 ± 0.24	5.1 ± 0.97	7.2 ± 0.87	8.9 ± 0.52
*Solanum melongena* L.	5.89 ± 0.22	8.43 ± 0.29	10.66 ± 0.19	6.22 ± 0.22	9.77 ± 0.39	12.33 ± 0.19	5.1 ± 0.55	6.67 ± 0.38	11.17 ± 0.19
*Solanum torvum* Sw.	4.44 ± 0.56	6.27 ± 0.63	7.44 ± 0.29	3.5 ± 0.40	5.76 ± 0.34	6.9 ± 1.45	3.9 ± 0.89	6.2 ± 1.12	7.1 ± 0.92

### 3.6 FTIR analysis

**Figure 4:**
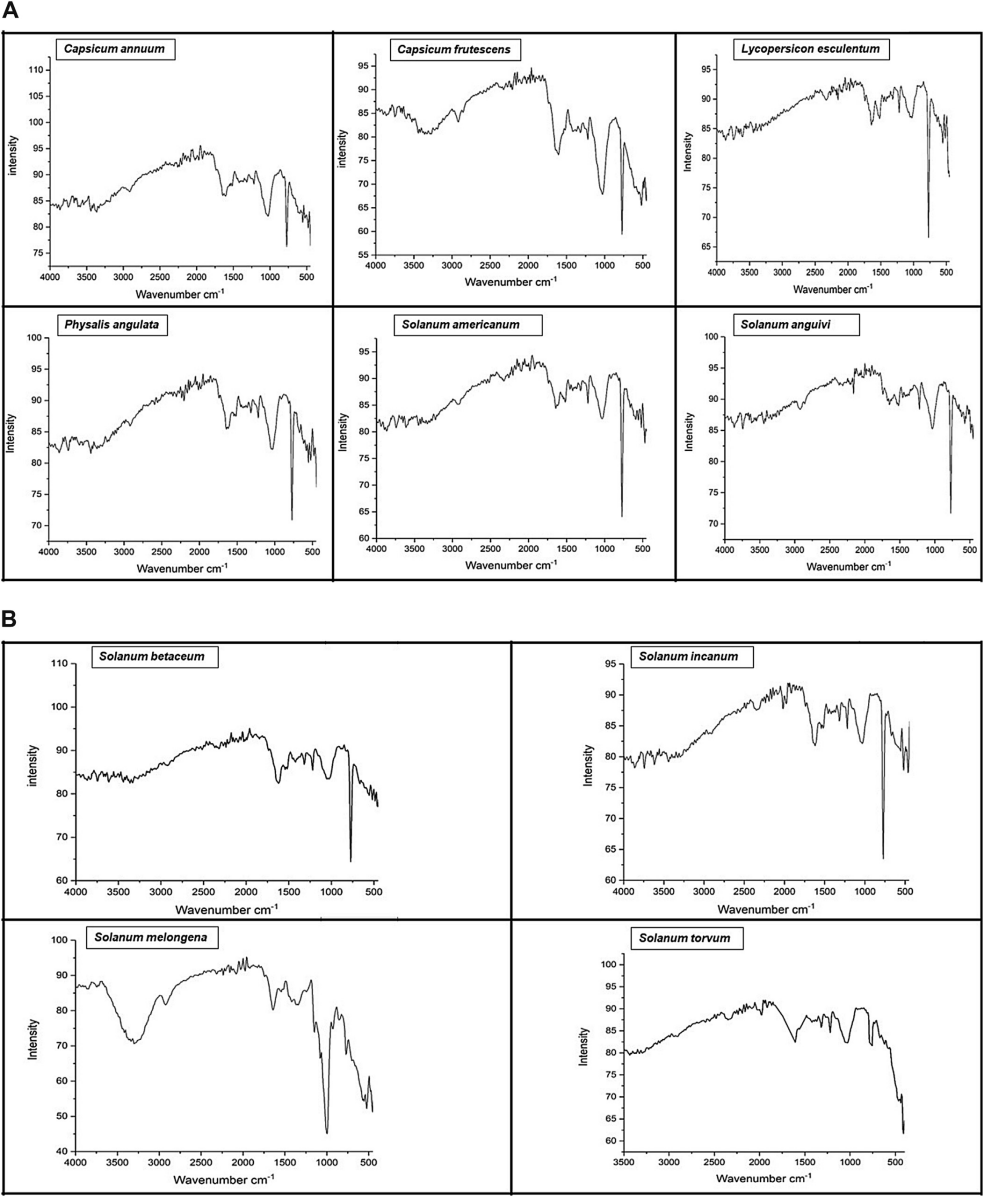
A. FT-IR spectra of Solanaceae plants. B. FT-IR spectra of Solanaceae plants.

The FTIR analysis showed the presence of different peaks indicating the presence of different functional metabolite groups in the plant extracts (Figure 4a and b).

### 3.7 Correlation between antioxidant DPPH scavenging activity, total phenolic and flavonoid content

**Figure 5:**
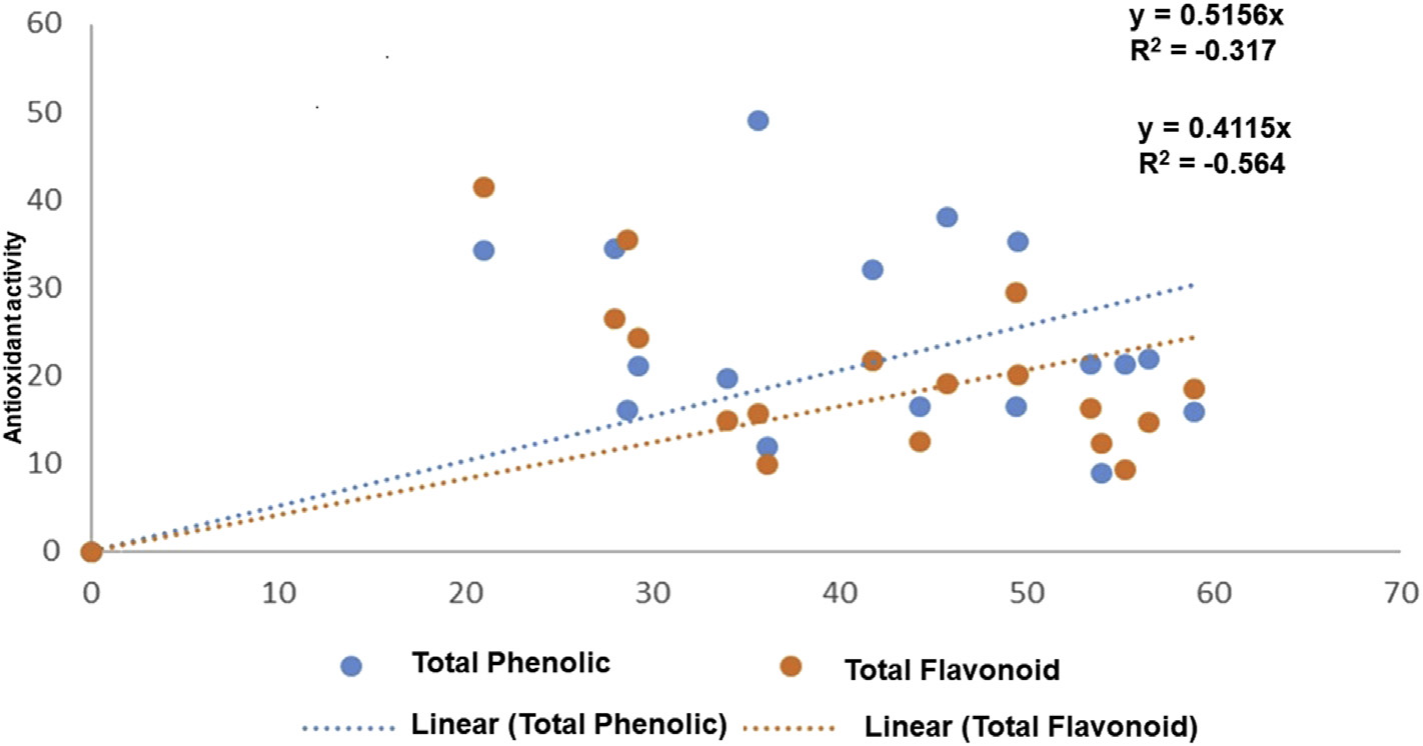
Correlation analysis between Antioxidant activity and Total Phenol and Flavonoid Content.

Due to their capacity to donate hydrogen atoms to free radicals, phenolic and flavonoid molecules are important antioxidant components that can deactivate these free radicals. A correlation analysis was performed for total phenol, flavonoid contents against antioxidant activities detected in Solanaceae plants species (Figure 5). A significant correlation between total phenol, total flavonoid content and antioxidant potential (y = 0.515x, R^2^ = 0.73 and y = 0.411x, R^2^ = 0.68, p ≤ 0.05 respectively) were observed at a 95% confidence level. It is reasonable to infer from the correlation coefficients (R-values) that the phenolic and flavonoid groups are primarily responsible for the antioxidant activity of the selected plant extracts. A correlation analysis was performed for total phenol and flavonoid contents against antioxidant activities detected in Solanaceae plants species (Figure 5). A significant correlation was observed between total phenol, total flavonoid contents and the antioxidant potential (y = 0.515x, R^2^ = 0.73 and y = 0.411x, R^2^ = 0.68, p ≤ 0.05 respectively). The strong correlation means that the phenolic and flavonoid contents contributed significantly to the antioxidant activity (Margaryan et al., 2017). The present analysis indicates that the antioxidant activity of studied samples is strongly correlated with high content of total phenolic and flavonoid that can play as reductones by donating electrons and reacting with free radicals thereby converting into more stable products (Margaryan et al., 2017).

## 4 Discussion

The present study shows that the Mizo people harbour significant knowledge on the traditional use of medicinal plants. In India, particularly in the north-eastern region, Asteraceae is the most dominant family of medicinal plants (Saklani and Jain, 1994). However, members of the Solanaceae family are very important medicinal plants used by the people of Mizoram (Khomdram et al., 2019). Local people not only collect medicinal plants but also collect a large number of wild edible fruits and vegetables to supplement their domestic nutritional requirements. They use different parts of these plants as medicine for different ailments where leaves and fruits are used for medicine preparation, in the form of a decoction, or as powders. The traditional knowledge of ethnobotanical uses of plants among the Mizo people requires documentation for preserving it for future generations. The present investigation will add significantly to the knowledge on the importance of Solanaceae plants that are used for various purposes.

The preliminary qualitative phytochemical analysis of edible plants of Solanaceae revealed the presence of various bioactive compounds which are reported to have different biological and therapeutic properties. Alkaloids are nitrogenous compounds having antioxidant potential and have been used in folk medicine (Quezada et al., 2006). Saponin is commonly used as a natural antioxidant and it has also been shown to promote apoptosis in tumour cells (Podolak et al., 2010; Bi et al., 2012). Tannins are well known antimicrobial agents (Sodipo et al., 1991), with antioxidant potential and have been used as active ingredients in medicine and beverages (Amarowicz and Troszynska, 2003). Likewise, flavonoids have antioxidant properties and have been reported to prevent cell damage, providing anticancer and anti-inflammatory activities (Salah et al., 1995; Okwu, 2004). Similarly, it has been reported that the presence of terpenoids influence antimicrobial properties (Mazher et al., 2016), and have been used as a protective agent against oxidative stress-induced diseases (Grassmann, 2005).

Plants are a diverse source of phenolic compounds with different functions and a majority are bioactive compounds with anti-cancer, anti-viral, antioxidant and anti-bacterial potentials (Manach et al., 2004). The total amount of phenol observed in the extracts was in comparison with the previous reports by Elekofehinti et al. (2013), Oyeyemi et al. (2015), Yousaf et al. (2013). Among the extracts, *S. anguivi* has the highest amount of phenol and this might be the reason that the plant is being used for the treatment of various skin diseases. Flavonoids are bioactive compounds belonging to the polyphenolic class and constitute the major antioxidant in fruits, plants and have advantageous effects on human health. Due to their high antioxidant properties, flavonoids are important sources of the human diet (Calado et al., 2015). They have high potential in antimicrobial, anticancer, anti-inflammatory and anti-allergic activities due to their ability to scavenge reactive oxygen species (ROS) consisting of free radicals (Montoro et al., 2005). In our study, the total flavonoid obtained was slightly higher than the previous reports (Hassan and Bakar, 2013; Mutalib et al., 2017; Vasco et al., 2009). Even a positive correlation of flavonoid and phenol content with a high antioxidant potential of the extracts was recognized (Table 4). Thus, the extracts, filled with high phenol and flavonoids, could be good sources of antioxidants thereby lowering the risk of diseases triggered by oxidative stress and also improving overall antioxidant capacity. Anthocyanins are involved in enzymatic reaction in the flavonoid biosynthesis pathway (Li et al., 2019). Anthocyanins also provide protection against certain chronic diseases such as hyperglycemia (Tsuda et al., 2003) and have been reported to inhibit the growth of tumour cells (Wang et al., 2013; Zhao et al., 2013), and improve vision (Kalt et al., 2014). Anthocyanins have high antioxidant potential, antibacterial properties and are used as natural food colorants (Naz et al., 2007). The total anthocyanin content was highest in *L. esculentum* (0.91 mg/g) followed by *P. angulata* (0.75 mg/g). *C. annuum* showed the lowest total anthocyanin content (Table 3). In our findings, the TAC was found higher than reported in previous works in *S. nigrum, S. tuberosum, S. lycopersicon, S. melongena, N. tabacum, P. hybrida* and *Withania somnifera* extracts (Kanungo et al., 2013; Wang et al., 2017). Recent reports have suggested that Solanaceae plants are promising resources for anthocyanin extraction (Li et al., 2019). The demand for anthocyanins is increasing in commercial industries and pharmaceuticals for the treatment of various diseases and also in beverage industries (Zhang et al., 2003). So, Solanaceae plants could be good sources of anthocyanins for various pharmaceutical and other commercial industries.

Antioxidants present in food are gaining prominence due to their significant function in maintaining human health by preventing diseases through inhibiting free radicals that are responsible for the spread of various diseases such as cancer, neurodegenerative disorders etc. The IC_50_ for DPPH of *L. esculentum* was lowest among the studied plants indicating strong antioxidant potential while *S. torvum* showed the highest DPPH. Phenols and flavonoids are multifunctional bioactive compounds that act as antioxidant, antimicrobial, anti-inflammatory and anti-cancer agents. Several studies have concluded that these multifunctional bioactive compounds are the major contributors to the antioxidant potential of plant extracts (Shahidi and Ambigaipalan, 2015). Hence, the free radical scavenging capacity observed in our study could be due to high levels of phenols and flavonoids in the extracts. This is in agreement with a report, showing higher free radical scavenging activity with higher overall phenolic and flavonoid contents (Zhang et al., 2016). Hence, the present study reveals that *L. esculentum* has a strong antioxidant potential. This property may be due to higher phenol, flavonoid and anthocyanin contents, which are required for scavenging activity, in *L. esculentum*. It is also known that the amount of phenolic and flavonoid contents in plants are responsible for the free radical scavenging activity. Our study suggests that the extracts of edible plants of Solanaceae display high antioxidant capacity. Environmental conditions like extreme temperature, water stress, and high light intensity can cause oxidative damage by over-production of toxic ROS (Bowler et al., 1992). However, plants can protect themselves against oxidative damage using their antioxidant systems such as anti-oxidative enzymes and non-enzymatic compounds (Mittler, 2002). Plants contain various anti-oxidative enzymes including SOD, CAT, APX etc (Wang et al., 2009). SOD converts superoxide radicals into hydrogen peroxide, APX uses ascorbate as an electron donor to reduce hydrogen peroxide to water and CAT dismutases hydrogen peroxide into water and oxygen (Wang et al., 2009). Living organisms can protect themselves from the toxic effects of ROS. SOD, APX and CAT are enzymes that help in detoxifying ROS. Increased level of SOD, APX and CAT can lead to enhanced oxidative stress protection (Gupta et al., 1993). Previous reports have also shown that Solanaceae plants have potential activities of SOD, APX and CAT (Yu et al., 1998; Tang et al., 2006; Kanungo et al., 2013). Our investigation confirms that the Solanaceae plants are good sources of SOD, APX and CAT that have significant value in reducing stress oxidative reactions. Owing to their high antioxidant capacity, these plants can serve as good sources of antioxidants in pharmaceutical and nutraceutical formulations.

The carbohydrate content in *S. torvum* (7.033 mg/g) was found to be much higher than the previous work reported by Akoto et al. (2015). The protein content in the plant extracts was also found to be higher than a previously reported value of 2.32 mg/g (Agoreyo et al., 2012). High values of protein and carbohydrate indicates rich in essential nutrients that could be utilized for enhancing nutrition. The mineral ion compositions of the plants were also relatively high in all the studied samples. Dietary intake of potassium has shown to have a significant effect on coronary heart diseases by reducing blood pressure (Weaver, 2013). Calcium is an essential mineral ion for the human diet and is involved in cell differentiation, muscle and bone formation (Roberts et al., 2000). Sodium is required for many physiological processes, body fluid balance and cellular homeostasis (Abdulrahman, 2004). Magnesium is essential for the circulatory system and is important for metabolism (Nwauzoma and Dawari 2013). Our study also showed the presence of micronutrients such as Fe, Cu, Mn, Zn. These micronutrients are required for crucial metabolic processes like respiration and DNA synthesis (Lieu et al., 2001). These findings supports effective utilization of these plants as a source of minerals or nutrient supplement.

Antibiotic resistance is an epidemic that continues to plague the healthcare system in both developing and developed countries around the world. The appearance and dissemination of multidrug-resistant pathogens have significantly jeopardized conventional antibacterial therapy. This has led to a hunt for new antimicrobial sources preferably from plants that contain various bioactive compounds with established therapeutic properties. The present study was undertaken to assess the antimicrobial efficacy of edible plants of Solanaceae against multi-resistant bacterial strains- *B. subtilis, E. coli and P. aeruginosa*. Results indicated that the plant extracts exhibited significant antibacterial activities towards the tested bacterial isolates. *L. esculentum* extract showed maximum activity against all the three pathogens. The inhibition was even higher than one reported on methanol extracts of other Solanaceae plants (Rawani et al., 2013). One of the most serious challenges to humanity is the rise of multidrug resistance by pathogens. The application of effective plant extracts might be a valuable option in combating this phenomenon and the plants studied in the current investigation could be useful in combating antidrug resistance for these tested bacterial strains. However, further investigations are sought to evaluate anti-viral, anti-fungal and anti-parasitic activities to harness the potentials of these plants.

Another important aspect of our study was to identify the functional groups found in these plant extracts using FTIR. This analysis helps in the identification of chemical composition, elucidation of the chemical structure and to understand the importance of functional groups as bioactive compounds for phyto-pharmaceutical formulations. The plants have shown similar infra-red spectrum and some intense bands at various frequencies which define the presence of O–H (hydroxyl), O–H stretch (carboxylic acid), O–H bend (phenol or tertiary alcohol) C–H stretch (alkanes), C=C–C (aromatic compounds), C=C stretch (ketone), N–O (nitro compound), C–O (ether), C–N (aromatic primary amines), N–H (amines), C≡C (carbonyl) and C–Br (aliphatic bromo compounds) (Table 7) groups. The presence of these functional groups indicates the presence of different metabolites such as aldehydes, alkanes, alkenes, alkynes, alkyl halides, aliphatic amines, primary and secondary amines, alcohols, aromatics, carboxylic acids, esters, ethers, glycogen, hydroxyl, lipid, organic halogen compounds, nitro compounds, phenols and triglycerides, that are integral parts of most of the secondary metabolites such as alkaloids, flavonoids, tannins, terpenoids and polyphenols (Poojary et al., 2015). Functional groups in the plants can be used in different pharmaceutical products such as for anti-cancers, anti-ulcers, jaundice, headache, stomach ache and anti-inflammatory drugs; or as sources of antimicrobial, antioxidant compounds etc (Baker, 1982; Skoog et al., 2007; Maobe and Nyarango, 2013). This may also be the reason why traditionally these plants are used by the locals in the treatment of stomach ache, as anti-inflammatory medicine etc (Table 1). The phytochemical screening and FTIR analysis showed that various bioactive compounds were found in these plant extracts that can be used as active antioxidant and anti-microbial agents. The current study also revealed clear discrimination between the plant parts tested (leaf, fruit, whole plant etc.), displaying significant heterogeneity for the identification of bioactive phytochemicals that can be used as herbal medicines. However, further studies are necessary to evaluate *in vivo* biological activities of the bioactive phytochemicals and for designing effective phyto-pharmaceutical formulations.

**Table 7:** Evaluation of FT-IR spectra of solanaceae plants.

Frequency range (cm^-1^)	Peak wavenumber (cm^-1^)										Functional group
3870–3550	*C. annuum* L.	*C. frutescens* L.	*L. esculentum* Mill.	*P. angulata* L.	*S. Americanum* Mill.	*S. anguivi* L.	*S. betaceum* Cav.	*S. incanum* L.	*S. melongena* L.	*S. torvum* L.	O–H stretch alcohol
3500–3200	3750	3672	3672.5	3865	3742	3865	3834	3741.9		3672	O–H stretch vibration presence of alcohols, phenols
3300–2850	3441	3441	3387	3441	3449	3649	3487	3417.9	3364	3325	O–H stretch vibration, carboxylic acids
2500–2300		2916		2924	2924	3225		3302.1	2924		C–H stretch vibration, alkenes
2260–2100			2330	2307			2446			2484	C=C stretch vibration, alkynes
1990–1739		2160	2152.6	2137	2207	2160		2237.4		2237	Ester C=O stretch, lipid, triglycerides
1700–1600		1836	1743.7		1983	1921	1844	1975.1		1975	C=C stretch vibration, alkenes
1550–1475		1605			1643	1651			1620		N–O asymmetric stretch, nitro compounds
1470–1400					1520			1543.1			C–C stretch vibration, aromatics
1400–1320			1458.2		1458	1458	1420			1458	N–O stretch vibration, nitro compounds
1300–1290		1319		1319					1319	1319	C–O stretch vibration, alcohol, carboxylic acids, esters, ether
1275–1150											C–H wag stretch vibration, alkyl halides
1020–1000		1219	1219	1219	1219	1219		1219	1242	1219	C–N stretch vibration, aliphatic amines
990–800		1026			1034	1034			1034	1034	N–H wag stretch vibration, primary & secondary amines
790–690											C (triple bond)C-HC-H bend stretch vibration, alkynes
680–510	772	772		771.5			77.5	741.53	779	772	C–Br stretch vibration, alkyl halides, glycogen
490–400	556	517	640.37	671.2	594.1	617	664	616.92	556	617	Halogen compound

## 5 Conclusions

Ethnobotanical uses, bioactive compound compositions, antioxidant activities, nutrient compositions and antimicrobial potential of edible plants of Solanaceae from Mizoram, India were analysed. These Solanaceae plants contain various bioactive phytochemicals, antimicrobial agents with various functional groups and have promising nutritional and antioxidant potential. Results demonstrated that these plants could be used as an easily accessible source of natural bioactive compounds with antioxidant and antimicrobial potentials and can also substitute synthetic drugs. To the best of our understanding, this is the first complete study of edible Solanaceae plants from Mizoram to investigate bioactive compounds, mineral nutrient contents, antimicrobial potential, antioxidant determination and identifying functional groups. Further studies on these plant species could open a new perspective for developing novel health-promoting agents in pharmaceutical and nutraceutical industries.

## Declarations

### Author contribution statement

Laldinfeli Ralte: Performed the experiments; Analyzed and interpreted the data; Contributed reagents, materials, analysis tools or data; Wrote the paper.

Usha Bhardwaj: Analyzed and interpreted the data; Contributed reagents, materials, analysis tools or data; Wrote the paper.

Y. Tunginba Singh: Conceived and designed the experiments; Analyzed and interpreted the data; Contributed reagents, materials, analysis tools or data; Wrote the paper.

### Funding statement

This research did not receive any specific grant from funding agencies in the public, commercial, or not-for-profit sectors.

### Data availability statement

Data included in article/supplementary material/referenced in article.

### Declaration of interests statement

The authors declare no conflict of interest.

### Additional information

No additional information is available for this paper.
